# Biomedical ethics and regulatory capacity building in Africa: the case study of São Tomé and Príncipe

**DOI:** 10.1080/11287462.2026.2698145

**Published:** 2026-07-22

**Authors:** Jorge P. B. Batista, Adionilde Aguiar, Vânia Castro, Jeryson Costa, Isaulina Barreto, M. Patrão Neves

**Affiliations:** a Portuguese Pharmaceutical Society, Lisbon, Portugal; b Unidade de Ensino e Investigação de Saúde Pública Global, Instituto de Higiene e Medicina Tropical, Universidade NOVA de Lisboa, Lisbon, Portugal; c National Health Ethics Committee for Scientific Research, São Tomé, São Tomé and Príncipe; d Pharmaceutical Department of the Ministry of Health, São Tomé, São Tomé and Príncipe; e Victor Sá Machado Higher Institute of Health Sciences, São Tomé, São Tomé and Príncipe; f Ministry of Health, Dr. Ayres de Menezes Central Hospital, São Tomé, São Tomé and Príncipe; g University of the Azores, Faculty of Humanities and Social Sciences, Ponta Delgada, Portugal

**Keywords:** Biomedical ethics, regulatory capacity building, São Tomé and Príncipe, clinical trials, legislative frameworks, Portuguese-Speaking African Countries (PSAC)

## Abstract

The Biomedical Ethics and Regulatory Capacity Building for Portuguese Speaking African Countries (BERC-Luso) Project aimed to strengthen biomedical ethics and regulatory capacity in Lusophone African Countries. At the legislative level, it promoted the alignment of national laws with international best practices for biomedical research. At the institutional level, it promoted the establishment and strengthening of National Regulatory Authorities (NRA) and National Ethics Committees (NECs). This project also included professional training to enhance national expertise; however, this article focused primarily on the legislative and institutional developments achieved in São Tomé and Príncipe. It examines how capacity building translated into national regulatory reform. At the project's onset, São Tomé and Príncipe had minimal legislative and institutional frameworks and no specific authorities or legislation in the field. Despite these shortcomings, political commitment and stakeholder engagement enabled major developments. The country established an NEC for scientific research, competent in clinical research oversight and advanced the legal and institutional proposal for an NRA, setting key building blocks for future clinical trials. These advancements underline the transformative potential of capacity-building programs in resource-limited settings. This experience may serve as a model for similar initiatives in countries seeking to strengthen ethical and regulatory frameworks for biomedical research.

## Background

São Tomé and Príncipe is one of the 5 Portuguese-speaking African countries (PSAC), together with Angola, Cabo Verde, Guinea-Bissau, and Mozambique, which integrated the Biomedical Ethics and Regulatory Capacity Building for Portuguese Speaking African Countries Project (BERC-Luso), financed by the European Development Clinical Trial Partnership (EDCTP), the Calouste Gulbenkian Foundation, and supported by the World Health Organization (WHO) and the United Nations Educational, Scientific and Cultural Organization (UNESCO). It lasted for 4 years, between October 2018 and September 2022.

The general aim of the project was to enhance biomedical ethics and regulatory capacities through a threefold action: legislative, institutional and professional. At the legislative level, the goal was (i) to study current legislation regarding biomedical research, particularly clinical trials; (ii) to compare it with international best practice requirements; (iii) to identify omissions; and (iv) to propose legislative revisions accordingly. At the institutional level, the goal was (i) to identify the existence of the National Regulatory Authority (NRA) and the National Ethics Committees (NEC), (ii) to promote their creation, where they did not exist, or to upgrade their status, where they exist at the initial level of a department in the Ministry of Health, and (iii) to promote a structure, organization and procedures adequately to their missions and in line with international requirements. At the professional level, the goal was (i) to provide basic education on the ethical and legal requirements for biomedical research in compliance with good international practice, (ii) to introduce them to effective practice through a training course for the ethical and regulatory evaluation of clinical studies, and (iii) to enable them to become trainers in the national scientific community, with a multiplier effect (Patrão Neves & Batista, 2022). In this context, “capacity” refers to the ability of national systems to develop and implement ethical and regulatory frameworks for biomedical research, including appropriate legislation, institutional oversight structures, and trained professionals. While BERC-Luso addressed these three dimensions, this article focuses primarily on the legislative and institutional developments and achievements in São Tomé and Príncipe. These two domains (legislative and institutional) provide the fundamental minimal requirements for conducting clinical trials and deliver a framework for specific training.

This threefold approach is essential for countries seeking to develop robust governance systems for biomedical research. Adequate and appropriate legislative frameworks, institutional oversight mechanisms, and expertly trained professionals are fundamental to ensuring that biomedical research, including clinical trials, is conducted ethically, transparently, and in accordance with established international standards. Strengthening these three domains can also support more active participation in international research collaborations, while safeguarding the rights, safety, and well-being of research participants. Furthermore, participation in international clinical research brings additional direct (e.g. access to innovative medicines and cutting-edge technologies) and indirect (e.g. establishment and associated industries and services) advantages (Strüver, 2017). To date, São Tomé and Príncipe has registered a negligible number of clinical trials, with only one study being published in the literature, more than 20 years ago (Gil et al., 2003). Without robust legislation, proficient institutions, and competent professionals, the basic conditions to attract and develop clinical trials are not in place.

In this process, São Tomé and Príncipe stood out as a country that made the most significant progress during the four-year project period. Its experience was selected as a case study and merits further reflection, so that it can be replicated with the necessary adaptations to other realities.

The broader impact of the BERC-Luso Project across the five Portuguese-speaking African countries has been previously evaluated and published elsewhere, demonstrating substantial progress in the legislative, institutional, and professional training domains (Batista et al., 2025). However, the mechanisms through which these capacity-building interventions translated into concrete national reforms remained underexplored. This article complements previous research and presents a focused case study of São Tomé and Príncipe—the country that achieved the most rapid legislative and institutional transformation during the project period—through its regulatory reform following capacity building.

## São Tomé and Príncipe country profile

The Democratic Republic of São Tomé and Príncipe is an archipelago of two Islands – São Tomé (850 km^2^) and Príncipe (300 km^2^) – located in the Gulf of Guinea in the Atlantic Ocean, about 380 km away from the west coast of Africa. Independent from Portugal since 1975, Portuguese has been São Tomé and Príncipe's official language. The population, which is mostly young (50% under 18), is around 231,856 (estimated INE 2024), with a natural growth rate of 2.0%, an average life expectancy of 69.2 years and a crude mortality rate of 5.3% (INE 2022). Its GDP is 1.3% (INE 2019).

With a tropical‒humid climate (28°C average annual temperature), it has dense and pristine rainforests, with rich and unique biodiversity (World Bank, 2024). A number of ethnopharmacological studies published elsewhere have been conducted in the country, leading to the identification of around 350 endemic plants of medicinal interest (Currais et al., 2014; Madureira et al., 2012). Furthermore, the Príncipe Island has been a UNESCO World Biosphere Reserve since 2012 (Biosphere reserves in Africa, 2024).

This snapshot of the country highlights some of its multiple vulnerabilities, such as its double territorial discontinuity, its separation from the African mainland and its dispersion on two islands. At the same time, there are factors considered as assets, such as the youth of the population and the rich biodiversity of the archipelago, which offers exciting potential for scientific research.

## The BERC-Luso project in São Tomé and Príncipe

BERC-Luso officially started in October 2018 and, at the time, did not include São Tomé and Príncipe (Patrão Neves and A Ribeiro, 2019). The multiple contacts made by the project's coordinators during the project's design and grant submission phase were never finalized due to communication difficulties and poor initial engagement. Owing to these blocking factors, an executive decision was made to submit the project for EDCTP funding with only 4 PSAC, thus excluding São Tomé and Príncipe. Despite not being formally involved, it was agreed by the project coordinator that informal involvement would be desirable in view of possible future formal consortium integration. After the official start of the project, it was possible to convene a meeting between representatives of the country's political power and the project's coordinators, which proved decisive for the inclusion of São Tomé and Príncipe in the BERC-Luso Project. For this approach to be successful, diplomatic ties were created, namely, through embassies, with the support of the country's Minister of Foreign Affairs.

This late integration into the project (officially only in February 2020) showed an initial disadvantage for the country. However, owing to political support and commitment (which turned out to be key for success), this delay was overturned, facilitated by the country’s immediate informal inclusion in the project’s workstreams.

### Pre-project legislative status

São Tomé and Príncipe's participation in BERC-Luso took place officially in two stages. First, a lawyer was appointed to an international legal team that surveyed the PSAC legislation on biomedical research and proposed recommendations to strengthen the regulatory framework. Second, in the appointment of five professionals from different scientific fields, with national roles in medicine management and ethical evaluation, identified the institutional structures in the country for future conduct of clinical trials.

The international legal team first identified the indispensable ethical and legal requirements for establishing an essential regulatory framework for the development of biomedical research. This was followed by a survey of the relevant national legislation. The results shown in Tables 1–3 highlight the paucity of legislative conditions for São Tomé and Príncipe (adapted from Martinho da Silva, 2019).

**Table 1.** Legislation and applicable guidelines to biomedical research (adapted and revised from Martinho da Silva et al., 2019).

Portuguese-speaking African country	Law identification (number, name, date)	Legislation context (in 2019)	Concrete provisions	Clinical trials (with medicinal products)	Other biomedical research
São Tomé and Príncipe	–	–	–	–	–
Angola	Law no. 21-B/1992, of 28 August	Basic law of the National Health System	Article 16 (Research), no. 1, 2	Article 21 (Clinical Trials of medicinal products)	–
	Presidential Decree no.180/2010, of 18 August	Basic Law of the National Pharmaceutical Policy	Article 16 (Medicines sample), no. 2	–	–
Cabo Verde	Law no. 41/IV/2004, of 5 April	Basic law of the National Health System	Article 22 (Research)	Yes	Yes
	Implementing Decree no. 23/2014, of 10 June	Approves the Statutes of the National Institute of Public Health (INSP)	Article 5 no. 1	Yes	Yes
	Resolution no. 5/2008, of 18 February	Approves the National Health Policy (2007)	VIII.8 Strategies for health research	Yes	Yes
Guinea-Bissau	In project	National Regulatory Authority of Medicines and Health Products (ARFAME, I.P.)	Article 4, no. 2, c) and d)	Yes	–
Mozambique	Law no. 12/2017, of 8 September	Law on medicines, vaccines, and other biological products for human use	Article 39 to 44 (Clinical trials—unregulated)	Yes	–
	Resolution no. 4/2017, of 26 May	Organic Statutes of the Ministry of Health	Article 10, no. 1 f)	–	–

**Table 2.** Independent validation and inspection of clinical trials (adapted and revised from Martinho da Silva et al., 2019).

Portuguese-speaking African country	Law identification (number, name, date)	Legislation/soft law context (in 2019)	Concrete provisions	Clinical trials (with medicinal products)	Other biomedical research	Mandatory character
São Tomé and Príncipe	–	–	–	–	–	–
Angola	–	–	–	–	–	–
Cabo Verde	Resolution no. 5/2008, of 18 February	National Ethics Commission in Health Research	VIII.8, no. 5	–	–	–
	Decree Law no. 26/2007, of 30 July	National Ethics Committee in Health Research	All	All health research	All health research	Yes
	Implementing decree no. 43/2014, of 10 June The	National Institute of Public Health as the national coordinating agency for health research in the country	Article 5, no. 1 a)	Yes	Yes	–
Guinea-Bissau	–	–	–	–	–	–
Mozambique	Law no. 12/2017, of 8 September	Law on medicines, vaccines, and other biological products for human use	Article 39, no. 1 and 2 Article 42 (no. 1 and 2) Article 43	Yes	–	–
	Internal document	National Bioethics Committee on Health	–	Yes	Yes	Yes (only in clinical trials)

**Table 3.** Checklist of ethical and regulatory requirements covered in existing legislation or in internal regulation (adapted and revised from Martinho da Silva et al., 2019).

Country	Primacy of the human being/dignity	Scientific approval	Ethical approval	Informed consent	Consent of people who cannot consent	Data confidentiality	Conflict of interest	Risk/benefit assessment	Safety and supervision
São Tomé and Príncipe	–	–	–	–	–	–	–	–	–
Angola	✓	✓	–	–	–	–	–	–	–
Cape Verde	✓	–	✓	✓	–	–		–	✓
Guinea-Bissau	–	–	–	–	–	–	–	–	–
Mozambique	–	–	✓	✓	–	✓	✓	✓	–

Legend: ✓ existent / – absent.

Table 1 shows the data obtained from a survey of the legal provisions in force applicable to biomedical research at the beginning of the project in each of the PSACs. It was therefore clear that São Tomé and Príncipe started the project without any legislation applicable to biomedical research and in a backward position compared to the other countries considered.

Table 2 identifies the legal existence of institutions with specific competencies to evaluate and monitor biomedical research in the respective country. It was therefore clear that São Tomé and Príncipe, like Angola and Guinea-Bissau, did not have institutions to ensure independent validation and inspection of biomedical research projects.

After surveying the legislation in force applicable to biomedical research in each PSAC, an analysis was made of their respective norms in order to check for some of the ethical and regulatory requirements. The nine selected requirements (Table 3) were identified on the basis of good research practices expressed in numerous international ethical and legal documents. Given the absence of legislation on biomedical research in S. Tomé and Príncipe, the minimum ethical and regulatory requirements identified to pursue it were not guaranteed in the country either.

In summary, in the legislative context in force in the PSACs, with respect to biomedical research, we registered that São Tomé and Príncipe was the only country with a total lack of conditions for independently conducting clinical trials.

In addition to a robust legislative framework, the second key factor for promoting clinical research is the existence of at least two indispensable institutions: a National Regulatory Authority (NRA) and a National Ethics Committee (NEC), both with independent oversight.

The national team of five professionals from S. Tomé and Príncipe first confirmed the absence of these two fundamental institutions in a form capable of independently overseeing clinical research. Nevertheless, the country already had a ‘Pharmaceutical Department of the Ministry of Health’, which carried out some specific functions of an NRA, and also a ‘Health Ethics Committee for Scientific Research’, created in 2018, which functioned as an ethics review body for general scientific research in health care, but lacked the mandate and technical capacity to review clinical study proposals; as well as a hospital ethics committee, an ethics body created to resolve professional ethical conflicts and support clinical practice: both institutions were not suitable to review scientific protocols for research projects. Furthermore, it was also confirmed that there was a lack of legislation to create NRA and NEC and regulations to frame the functioning of existing institutions, according to international standards within the biomedical research field. It was confirmed that only draft legislative texts existed, without being subject to public consultation and discussion yet.

Moreover, it was verified that no law was enacted to conduct clinical trials, nor was this activity regulated or implemented in the country.

### Pre-project institutional status

Table 4 describes the institutional framework at the time of the legislative comparative study (September 2019, adapted from Martinho da Silva et al., 2019) for specific and fundamental institutions on biomedical research oversight. In general, this review demonstrated that there was significant dispersion in the legal framework within the PSAC: some countries had enacted a general law to regulate clinical trials, others had opted to issue Decree–Laws, and still others had chosen Resolutions, or other means of law enforcement. Multiple legislative instruments have also been enacted to establish a national competent authority, approve statutes and bylaws, and other regulatory texts. The review showed an absence of a harmonized framework, which led to inconsistent application of the law, complicated enforcement, and made the Lusophone region in Africa less attractive for the implementation of research projects.

**Table 4.** Institutional Framework (September 2019, adapted and revised from Martinho da Silva et al. (2019)).

Country	National Regulatory Authority (NRA)			National Ethics Committee for Clinical Research (NEC)			Legislative Framework to independently conduct Clinical trials
	Name of institution	Applicable Legislation	Internal Regulation for Clinical Trials	Name of institution	Applicable Legislation	Internal Regulation	
São Tomé and Príncipe	Pharmaceutical Department in the Ministry of Health **●** **○○**	Decree-Law nr 86/2009	Non-existent	Non-existent	Non-existent	Non-existent	Absent
Angola	National Directorate of Medicines and Equipment of the Ministry of Health **●●** **○**	Presidential Decree nr 180/2010 of August 18	Non-existent	Ethics Committee of the National Institute for Health Research	Non-existent	Non-existent	Present
Cabo Verde	Agency for the Regulation and Supervision of Pharmaceutical and Food Products **●●●**	Decree-Law nr 42/2004, of October 18	Non-existent	National Research Ethics Committee for Health	Decree-Law nr 26/2007 of July 30	Existent	Absent
Guinea-Bissau	Directorate of Pharmaceutical Services of the Ministry of Public Health, Family and Social Cohesion **●●** **○**	Decree nr 11/2010	Non-existent	National Health Ethics Committee—National Institute of Public Health	Decree nr 12/2010	Non-existent	Partially absent
Mozambique	National Pharmacy Directorate of the Ministry of Health **●●** **○**	Resolution nr 4/2017 of May 26	Non-existent	National Bioethics Committee for Health	Ministerial Order 58/2017 of August 31, 2017	Existent	Present

Legend: institutional maturity level.

●○○ – Department in the Ministry of Health /●●○ – National Directorate of the Ministry of Health / ●●● – Independent National Regulatory Authority.

The survey of NRAs and NECs in the PSACs led us to highlight Cabo Verde as the only country with these two legally constituted institutions. Once again, in comparative terms among the PSACs, São Tomé and Príncipe showed the lowest level of institutional organization maturity.

### Goals

In São Tomé and Príncipe, the project pursued two interrelated goals. First, it aimed to support the development of an ethical and legislative framework for biomedical research, in general, and the conduct of clinical trials in particular. Second, it sought to contribute to the formal establishment and regulation of the two key overseeing institutions required for independent biomedical research governance: a National Regulatory Authority (NRA) and a National Ethics Committee (NEC). These goals reflected the country’s starting position among the PSAC, characterized by limited or absent legislative provisions, incomplete institutional presence, and the absence of a specific framework for clinical research oversight.

### Methodology

#### Methodology for building legislative and institutional development

The goals were pursued in alignment with internationally recognized ethical and regulatory standards for biomedical research, in order to support the development of a legitimate and credible national framework for future biomedical research, including clinical trials. These international ethical and regulatory standards for biomedical research included the Declaration of Helsinki, the Council of Europe Convention on Human Rights and Biomedicine (Oviedo Convention) and its additional protocol concerning biomedical research, UNESCO’s Universal Declaration on Bioethics and Human Rights, the Council for International Organizations of Medical Sciences (CIOMS) International Ethical Guidelines for Health-related Research Involving Humans, and the International Council for Harmonisation of Technical Requirements for Pharmaceuticals for Human Use (ICH) Guideline for Good Clinical Practice (ICH-GCP) (Council for International Organizations of Medical Sciences (CIOMS), 2016; Council of Europe, 1997, 2005; International Council for Harmonisation of Technical Requirements for Pharmaceuticals for Human Use (ICH), 2016; UNESCO, 2005; World Medical Association, 2013).

The methodology combined two complementary approaches: a top-down strategy and a bottom-up strategy. The top-down approach involved direct engagement with political, governmental, and legislative actors, including the Ministry of Health, national parliament, and other relevant public authorities, in order to raise awareness, support legal drafting processes, and promote institutional reform. In parallel, the bottom-up approach focused on empowering national professionals working in ethics, medicine regulation, and health governance through targeted training and project participation. This was reinforced through follow-up activities, enabling them to act as national advocates for system reform and implementation. Establishing a solid foundation to strengthen the regulatory framework for biomedical research in the country involved both meetings and liaisons with experts and participation in media actions (e.g. television programs, interviews) with message building for the general public. These actions were instrumental in designing and discussing legislation at the political level, which preceded its adoption and implementation, and advocated for institutional reinforcement.

#### Evaluation of implementation

To assess legislative and institutional developments associated with the BERC-Luso Project, the project coordination team developed a descriptive evaluation framework covering seven key domains relevant to biomedical research governance: the National Regulatory Authority maturity level, National Regulatory Authority Statutes, National Regulatory Authority Internal Regulations, National Ethics Committee maturity level, National Ethics Committee Statutes, National Ethics Committee Internal Regulations, and Clinical Research legislation. Each domain was assigned a score based on predefined institutional development criteria (detailed in Annex 1), ranging from the absence of regulatory structures to the establishment of fully operational institutions or legal frameworks. This framework was designed to provide a structured representation of legislative and institutional development associated with the project over time rather than to serve as a validated quantitative measurement instrument. Notwithstanding, the framework is conceptually aligned with maturity- and governance-based assessment frameworks used in health system and regulatory capacity evaluations, such as the WHO Global Benchmarking Tool for national regulatory systems and broader health system governance assessment frameworks (Global Benchmarking Tools, 2026; World Health Organization, 2010). Scoring was conducted by consensus jointly by the project coordination team and the national São Tomé and Príncipe team, based on documentary evidence of legislative and institutional changes. The results of this descriptive framework are presented in Figure 2 in the Results section.

### Implementing actions

**Figure 1.**
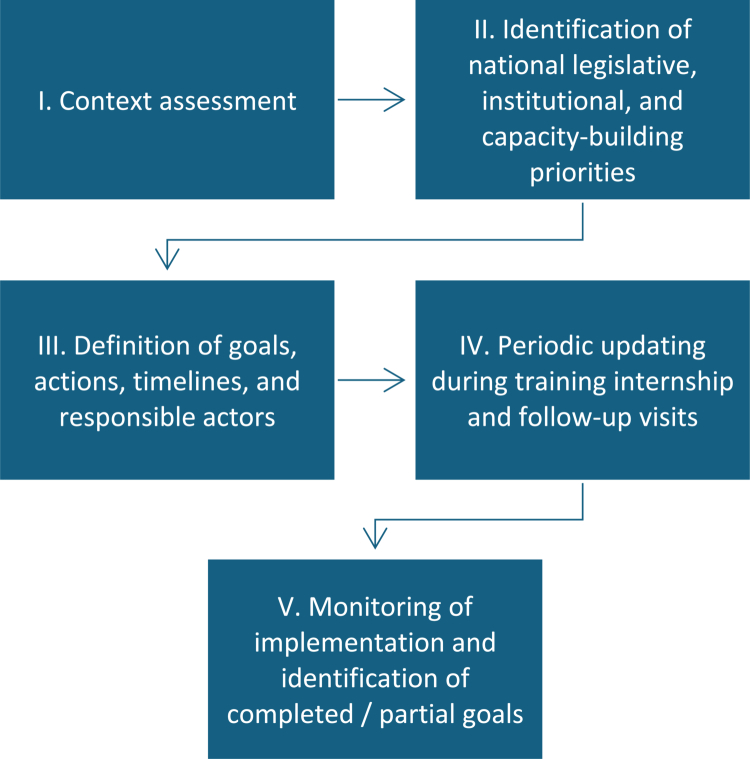
Main components of the Declaration of Commitment in São Tomé and Príncipe. The Declaration of Commitment served as a strategic country-level roadmap used to define priority actions, timelines, and responsible actors and was updated during the implementation of the project to monitor progress and refine objectives.

In the second year of the project (February 2020), as part of an educational theoretical training course, the Santomean team — like the other four PSAC teams – prepared its “Declaration of Commitment”. The Declaration of Commitment was a strategic planning document developed by each participating country team that outlined priority legislative, institutional, and capacity-building actions and targets to be pursued during the project, together with timelines and responsible stakeholders. It defined the objectives to be achieved, considering the legislative and institutional framework of the country and the specificities of each partner, and identified the necessary means for implementation. The Declaration of Commitment functioned as a practical country-level roadmap: it translated the broad objectives of the project into concrete actions, timelines, and responsibilities. This was instrumental in facilitating follow-up, strengthening accountability, and promoting the periodic revision of priorities in a flexible approach. For this reason, it may be considered a useful operational tool for comparable capacity-building interventions in resource-limited settings (Figure 1).

This “Declaration of Commitment” was updated in September 2021, during the practical training internship, and once again in February 2022, during a technical follow-up visit of the project’s Coordination Team to the country. These successive updates involved (i) an intermediate assessment of the implementation of proposed actions and the adequacy of the means used, (ii) the definition of new deadlines for pending actions, and (iii) the establishment of new, more ambitious goals, actions, and objectives based on those already achieved and fine-tuning of the methods used in both circumstances.

The goals (aims) detailed in the “Declaration of Commitment” (roadmap) were structured in alignment with the three dimensions of the BERC-Luso Project: legislative, institutional, and professional. Therefore, the roadmap categorized actions into three main areas: legislative actions (such as the approval of NRA statutes, internal regulations, and legislation or regulatory supervision of clinical trials); capacity-building actions (including the development of training programs for professionals and opportunities for twinning programs); and institutional actions (such as technical improvements in institutional procedures and the creation of ministerial-led programs at the national level to support clinical trials, including pharmacovigilance and inspections). The Santomean Declaration of Commitment included 3 legislative goals, 3 capacity-building goals, and 8 institutional goals. By the end of the project, 13 goals had been fully completed, and one goal had been partially completed, with no goals left incomplete. The evaluation of these goals is published elsewhere (Batista et al., 2025).

## Results

Key developments unfolded in sequence over a relatively short period. In February 2020, following the initial BERC-Luso training activities, the proposal for a dedicated National Ethics Committee (NEC) gained formal momentum. This was followed by the initiation of parliamentary discussions on the Internal Regulations of the future National Regulatory Authority, supported by the political momentum generated through the project. In 2022, the Minister of Health approved the Health Ethics Committee for Scientific Research (CESIC) by the Ministerial Order. In 2023, its internal regulations were approved. Owing to the national elections that followed, the Diploma that created the Statute of the National Regulatory Authority (ARFAMED) was not approved. However, there was a second submission to the new political power, namely, to the Council of Ministers for the approval of 3 diplomas: (i) Decree–Law, which creates ARFAMED and its statute; (ii) Medicine Diploma, which includes aspects related to clinical trials and medicine registration in the country; and finally (iii) Decree–Law, which approves the Legal Framework for Pharmacies and Medicine Sales Points. The clinical research framework was discussed, with additional political and legislative activity continuing into 2024.

Table 5 describes the institutional framework analysis following the conclusion of the BERC-Luso Project, which highlights significant developments in São Tomé and Príncipe. The country progressed from a Pharmaceutical Department within the Ministry of Health towards the formal establishment of a National Regulatory Authority, with the relevant legal framework submitted for governmental approval. By the end of the project and the subsequent follow-up period, legislation and internal regulations relevant to clinical trials – previously absent – had been proposed, approved, or advanced through formal institutional processes. Regarding the National Ethics Committee, a new Health Ethics Committee for Scientific Research was created, with its internal regulations approved as a direct outcome of the project. Furthermore, a legislative framework for the independent conduct of clinical trials was proposed in Law, with discussions held by political parties.

**Table 5.** Institutional framework (September 2024).

Country	National regulatory authority (NRA)			National ethics committee for clinical research (NEC)			Legislative framework to independently conduct clinical trials
	Name of institution	Applicable legislation	Internal regulation for clinical trials	Name of institution	Applicable legislation	Internal regulation	
São Tomé and Príncipe	São Tomé and Príncipe Pharmaceutical Sector Regulatory Authority (ARFAMED) **●●●**	Submitted for approval to the Council of Ministers	Proposed	Health Ethics Committee for Scientific Research (CESIC)	Order nr 01/GMS/2022	Existent	Proposed law
Angola	Medicines and Health Technologies Regulatory Agency (ARMED) **●●●**	Presidential Decree nr 136/2021 of June 1	Proposed	Ethics Committee of the Ministry of Health	New Organic Statute of the Ministry of Health (Presidential Decree nr 277/20 of October 26)	Proposed	Present
Cabo Verde	Independent Health Regulatory Authority (ERIS) **●●●**	Decree-Law nr 03/2019, of January 10	Non-existent	National Research Ethics Committee for Health	Decree-Law nr 26/2007 of July 30	Existent	Proposed framework
Guinea-Bissau	National Regulatory Authority for Pharmacies and Medicines (ARFAME). **●●●**	Existent	Non-existent	National Health Ethics Committee – National Institute of Public Health	Existent	Non-existent	Partially absent
Mozambique	National Medicines Regulatory Authority (ANARME) **●●●**	Decree nr 115/2020 of December 31	Present	National Bioethics Committee for Health	Ministerial Order 58/2017 of 31 August 2017	Proposed	Present

Legend: institutional maturity level.

●●● – Independent National Regulatory Authority.

**Figure 2.**
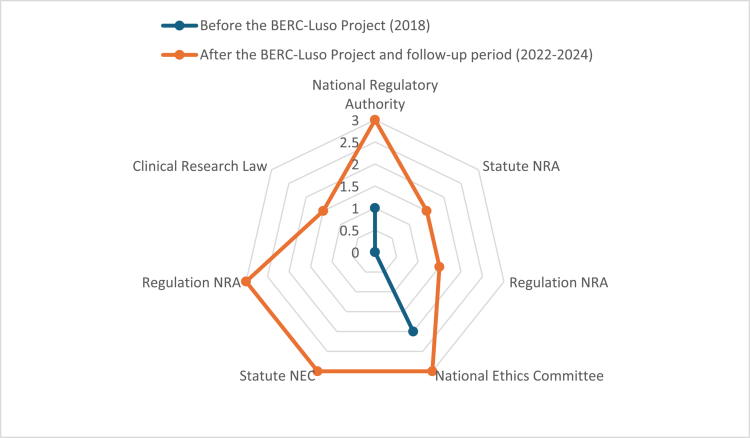
Evolution of the legislative and institutional framework for biomedical research in São Tomé and Príncipe, 2018–2022.

Figure 2 maps out the country’s development and the impact of BERC-Luso across different areas, including the adoption of legislation, the establishment of institutions, and the proposal of frameworks. This illustrates legislative and institutional evolution across key domains of biomedical research governance. Using the descriptive framework presented in the Methods section, São Tomé and Príncipe demonstrated substantial progress across all evaluated domains between the start of the project and the post-project follow-up period. Furthermore, the transformation initiated by the BERC-Luso Project (in 2018) continued even after the project's completion (in 2022). Between 2022 (the end of the BERC-Luso Project) and 2024 (the onset of CT-Luso Project), additional legislative initiatives were introduced, and institutional procedures continued to be strengthened. Through technical follow-up, continued political discussion, the resubmission of legal texts, and institutional strengthening under a successor project (CT-Luso), São Tomé and Príncipe continued to build capacity in this area (CT-Luso, 2025). Table 6 summarizes the evolution of the legislative and institutional framework following the BERC-Luso project and during the subsequent follow-up period extending to 2024.

**Table 6.** Impact of the BERC-Luso Project in the legislative and institutional framework (September 2024).

Settings	Before the BERC-Luso Project (2018)	After the BERC-Luso Project and follow-up period (2022–2024)
National Regulatory Authority (maturity)	Department in the Ministry of Health	Independent National Regulatory Authority
National Regulatory Authority (Statutes)	Statutes not approved	Statutes Proposed/under discussion
National Regulatory Authority (Internal Regulations)	Internal Regulations not approved	Internal Regulations Proposed/under discussion
National Ethics Committee (maturity)	Ethics Committee for General Research	National Ethics Committee for Clinical Research
National Ethics Committee (Statutes)	Statutes not approved	Statutes approved
National Ethics Committee (Internal Regulations)	Internal Regulations not approved	Internal Regulations approved
Clinical Research Law	Clinical Research Law inexistent	Clinical Research Law proposed/under discussion

Attention should be given to the area of the development of the National Regulatory Authorities in the different PSACs. As shown in Table 5, all partner countries increased their maturity level of the NRA to an independent National Regulatory Authority, a key component for the implementation of biomedical research projects in the country. This progress is particularly noteworthy as the project was sustained during the COVID-19 pandemic, which acted as a catalyst to this development.

## Conclusion

The BERC-Luso Project contributed to meaningful legislative and institutional development in Portuguese-speaking African countries and was particularly effective in São Tomé and Príncipe over a relatively short period. Importantly, the project also fostered increased awareness among national professionals (particularly those working in the pharmaceutical sector) of the scope and complexity of the ethical and legal requirements needed to enable the conduct of clinical trials. It further contributed to a clearer understanding of the potential benefits for the country in becoming an active partner in international biomedical research—an area that was not widely recognized prior to the project's implementation.

At the beginning of the project, São Tomé and Príncipe lacked a dedicated legislative framework for biomedical research and did not have the key oversight authorities required for the independent conduct of clinical research, in contrast to the other Portuguese-speaking African countries involved in the project. During (and following) the project, the country established the National Ethics Committee for Scientific Research, approved its internal regulations, and advanced the legal and institutional basis for a National Regulatory Authority, with a broader clinical research framework. Although some elements remained under discussion or pending official approval and enactment, the project succeeded in establishing important legislative and institutional foundations for future biomedical research oversight and for the eventual independent conduct of clinical trials in the country.

The São Tomé and Príncipe experience illustrates how capacity-building interventions can support national reforms even in settings that initially faced substantial structural and institutional limitations. As a focused national case study, this article shows how the legislative and institutional dimensions of capacity building may generate durable effects even beyond the formal project timeline and may offer useful lessons for similar initiatives in other resource-limited settings.

## Annex 1: Points attributed to each development area based on a standardized criteria:

National Regulatory Authority (maturity).

**Table:**

Criteria	Points
No Authority	0
Department in the Ministry of Health	1
National Directorate of the Ministry of Health	2
Independent National Regulatory Authority	3

National Regulatory Authority (Statutes).

**Table:**

Criteria	Points
Statutes not approved	0
Statutes proposed/under discussion	1.5
Statutes approved	3

National Regulatory Authority (Internal Regulations).

**Table:**

Criteria	Points
Internal Regulations not approved	0
Internal Regulations proposed/under discussion	1.5
Internal Regulations approved	3

National Ethics Committee (maturity).

**Table:**

Criteria	Points
No Authority	0
Ethics Committee in Hospital	1
Ethics Committee for General Research	2
National Ethics Committee for Clinical Research	3

National Ethics Committee (Statutes).

**Table:**

Criteria	Points
Statutes not approved	0
Statutes proposed/under discussion	1.5
Statutes approved	3

National Ethics Committee (Internal Regulations).

**Table:**

Criteria	Points
Internal Regulations not approved	0
Internal Regulations proposed/under discussion	1.5
Internal Regulations approved	3

Clinical Research Law.

**Table:**

Criteria	Points
Clinical Research Law inexistent	0
Clinical Research Law proposed/under discussion	1.5
Clinical Research Law approved	3
